# Transient regulatory T-cell targeting triggers immune control of multiple myeloma and prevents disease progression

**DOI:** 10.1038/s41375-021-01422-y

**Published:** 2021-09-28

**Authors:** Julia Dahlhoff, Hannah Manz, Tim Steinfatt, Julia Delgado-Tascon, Elena Seebacher, Theresa Schneider, Amy Wilnit, Zeinab Mokhtari, Paula Tabares, David Böckle, Leo Rasche, K. Martin Kortüm, Manfred B. Lutz, Hermann Einsele, Andreas Brandl, Andreas Beilhack

**Affiliations:** 1 Department of Internal Medicine II, University Hospital Würzburg, Würzburg, Germany; 2 Center for Interdisciplinary Clinical Research, University of Würzburg, Würzburg, Germany; 3 Graduate School of Life Sciences, University of Würzburg, Würzburg, Germany; 4 Mildred Scheel Early Career Center, University Hospital of Würzburg, Würzburg, Germany; 5 Institute for Virology and Immunobiology, Würzburg University, Würzburg, Germany

**Keywords:** Tumour immunology, Immunotherapy, Immunosurveillance

## Abstract

Multiple myeloma remains a largely incurable disease of clonally expanding malignant plasma cells. The bone marrow microenvironment harbors treatment-resistant myeloma cells, which eventually lead to disease relapse in patients. In the bone marrow, CD4^+^FoxP3^+^ regulatory T cells (Tregs) are highly abundant amongst CD4^+^ T cells providing an immune protective niche for different long-living cell populations, e.g., hematopoietic stem cells. Here, we addressed the functional role of Tregs in multiple myeloma dissemination to bone marrow compartments and disease progression. To investigate the immune regulation of multiple myeloma, we utilized syngeneic immunocompetent murine multiple myeloma models in two different genetic backgrounds. Analyzing the spatial immune architecture of multiple myeloma revealed that the bone marrow Tregs accumulated in the vicinity of malignant plasma cells and displayed an activated phenotype. In vivo Treg depletion prevented multiple myeloma dissemination in both models. Importantly, short-term in vivo depletion of Tregs in mice with established multiple myeloma evoked a potent CD8 T cell- and NK cell-mediated immune response resulting in complete and stable remission. Conclusively, this preclinical in-vivo study suggests that Tregs are an attractive target for the treatment of multiple myeloma.

## Introduction

Continuous advances in multiple myeloma (MM) therapy have improved the 5-year survival from 25% to over 50% over the last four decades [1]. Current developments of targeted humoral and cellular immunotherapies appear promising to further improve patient outcomes [2]. However, MM remains largely incurable urging the need for novel strategies to treat the disease [3, 4]. Strategies to overcome tumor immune escape will rely on a better understanding of the functional impact of the immune contexture in MM [5]. It has become clear that the bone marrow (BM) environment, the primary location of MM growth, makes it exceptionally difficult to tackle MM [6, 7]. The BM comprises several niches; some of them are highly immune-suppressive areas, perfectly suited for long-living stem cells and memory T and B cells [8]. The magnitude of this immune-suppressive capacity was impressively demonstrated as CD4^+^FoxP3^+^Tregs prevented the rejection of even MHC-mismatched allogeneically transplanted hematopoietic stem cells in immunocompetent hosts [9]. Tregs have been widely recognized as crucial players in tumor immune escape and disease progression in many types of cancer [10]. However, in MM the role of Tregs has remained controversial as Treg frequencies and function were reported as normal [11] or altered and dysfunctional in patients with MM [12–14] and to correlate with disease outcome [15]. Yet, recent work revealed myeloma cells directly induce and expand mouse and human Tregs in co-culture experiments in a contact-dependent and independent manner [16–18]. However, the precise mechanisms of how Treg-mediated immune suppression leads to the progression of MM within the BM microenvironment remain poorly understood. To this end, we employed two independent syngeneic MM models in immunocompetent mice [19, 20]. Consistently, Tregs in the BM of MM diseased mice displayed an activated phenotype in both models. In the BM Tregs did not homogenously distribute in myeloma-bearing mice but densely accumulated in areas of MM proliferation. By selectively depleting Tregs in DEREG mice at different time points of disease progression we show that Tregs not only were crucial for the initial hematogenous dissemination of MM cells in the BM, but also for the progression of MM during established disease. Strikingly, already a short and transient disruption of the Treg mediated suppression resulted in complete and stable remission of MM reducing the risk for autoimmunity and inaugurating Tregs as a target for MM therapy. Finally, we identified endogenous NK and CD8 T cells as the major effector cells responsible for the profound remission after Treg depletion.

## Methods

### Ethics statement

Experiments with human BM aspirates were approved by the Institutional Ethics Committee of Würzburg University Hospital. Written informed consent was obtained from all MM patients and all clinical investigations were conducted in accordance with the Declaration of Helsinki. All experiments with mice were approved by the Bavarian government (Regierung von Unterfranken). Mouse breeding and housing were under controlled conditions in the animal facility of the center of experimental molecular medicine (ZEMM), University Würzburg, with constant night/day cycles, temperature, and humidity in individually ventilated cages.

### Tumor models

The MOPC-MM mouse model was used as described before [19, 21]. 2 × 10^5^ MOPC-cells (MOPC-315.Luc-GFP.BMP3 cells, negative tested for mycoplasma contamination) were injected in the tail vein of female BALB/c mice (Charles River, Sulzfeld, Germany) or C.B6-Tg(Foxp3-DTR/EGFP)23.2Spar [22] (C.DEREG) positive or negative littermates (initially given by Tim Sparwasser and further bred in-house). Disease progression and tumor burden were monitored with bioluminescence imaging (BLI) starting 14 days after tumor cell injection as described [21, 23]. The average radiance of an ROI covering each mouse from the snout to the tailhead was analyzed with living image software (Perkin Elmer, Hopkinton, USA), values for dorsal and ventral view were added and normalized to the values of individual mice at the start of treatment. Transplantable Vk12653 cells [20, 24] (initial aliquot kindly given by Martha Chesi) were obtained from a splenic tumor and aliquots were frozen (complete cell isolate from tumor-bearing spleen ~33% tumor cells). 2 × 10^5^ cells were injected into the tail vein of C57BL/6JTyrc − 2 J/Foxp3.Luci.DTR-4 [25] (B6a.FoxP3.Luci.DTR, which had had been backcrossed from C57BL/6.Foxp3.Luci.DTR-4 [26] into a C57Bl/6 albino background; depletion of Tregs) or in wild-type littermates (MM control). Tumor progression was measured with serum protein electrophoresis (SAS-MX10, Helena Bioscience, Gateshead, UK) and the detection of M-spike. Tumor burden was assessed with flow cytometry at the endpoint of the experiment.

### Treg depletion experiments

For Treg depletion experiments C.DEREG [22] or B6a.FoxP3-Luci-DTR [26] mice were used. Both strains express the diphtheria toxin receptor under the FoxP3-promoter. By intraperitoneal administration of diphtheria toxin (20 µg/kg) on two consecutive days, Tregs are depleted efficiently. Tregs were either depleted before tumor cell injection or in established tumor mice that showed BLI signal (MOPC) or when M-protein (VK*MYC) was detected in most mice.

### Antibody-mediated depletion

CD4 cells (GK1.5; 200 µg) CD8 cells (YTS 169.4; initially 400 µg, for ongoing depletion 200 µg once weekly) asialo-GM1 (polyclonal; 35 µg), CD25 (PC61; 100 µg) or a Rat IgG2 (200 µg) were intraperitoneally injected in MOPC-MM mice with distinct BLI signal to deplete potential effector cells in-vivo. Treatment was started simultaneously with the administration of DTx, and effector cells were kept depleted by weekly injections.

### Flow cytometry analysis

1 × 10^7^ BM cells were blocked (10% normal rat serum) and stained (list of antibodies in Table S1) in deep well plates. Tregs were defined either by GFP expression (DEREG mice), or by intracellular staining of FoxP3. For the analysis of granzyme B expression, 1 × 10^7^ BM cells were incubated with Dynabeads mouse T-activator CD3/CD28 (ThermoFisher) and GolgiStop (BD Biosciences Heidelberg, Germany) for 4 h in RPMI at 37 °C before intracellular staining of granzyme B. All experiments were analyzed on an Attune NxT flow cytometer (Invitrogen) and FlowJo (version10.6 Tree Star, Ashland, USA) was used.

### MM patient samples

A total of 55 myeloma patients at different stages of disease were included in this study. BM aspirates were taken for MRD diagnosis and excess cells were processed for flow cytometry analysis. Erythrocytes were lyzed (Bulk-Lysis, Cytognos, Salamanca, Spain) and cells were stained similar to murine samples.

### Immunofluorescence microscopy

Bones of the hind legs of MM mice were isolated and processed as described [27]. For imaging, the Zeiss microscope (Imager.Z1m) was used. Analysis of images was done with ImageJ1.52j. The brightness of single channels was adjusted linearly, and the background was subtracted. CD4 T-cells positive and negative for GFP were counted manually and density was calculated. Stitching of single images to a larger file was also done with ImageJ using a plugin for stitching [28].

Additional methods are available in the supplementary information.

## Results

### Tregs accumulate within the bone marrow MM microenvironment

**Fig. 1:**
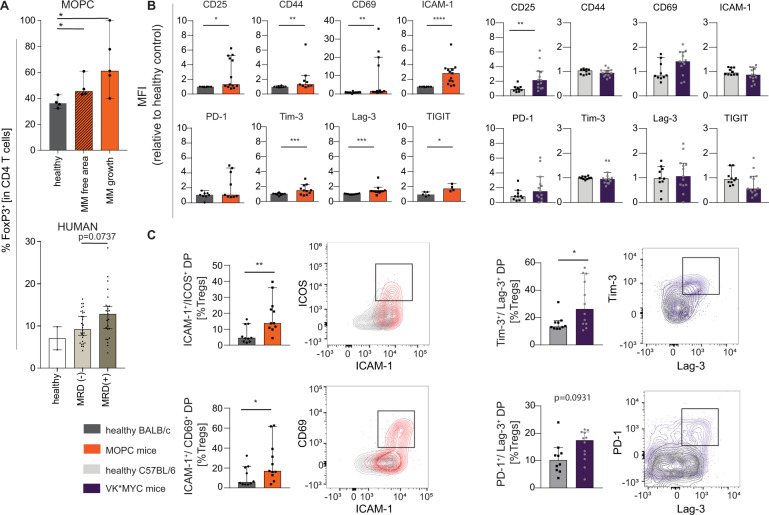
Tregs are highly abundant in areas of myeloma tumor growth and display an activated phenotype. **A** Frequency of Tregs was highest in mice with MOPC-MM in areas of tumor growth (*n* = 3–4, determined with immune fluorescence microscopy, cells were counted manually using ImageJ), and in bone marrow aspirates of MRD positive (*n* = 25) myeloma patients, compared to patients in complete remission (MRD negative) (*n* = 30), *n* = 2 healthy controls (determined with flow cytometry). **B** Change of mean fluorescence intensity of activation marker on Tregs from the BM of MOPC-MM mice (orange) or VK*MYC-MM mice (violet) relative to respective healthy controls (grey) analyzed by flow cytometry. Tregs from the BM of MOPC-MM mice (left) had significantly elevated levels of the T-cell activation markers CD25, CD44, CD69, and ICAM-1 whereas Tregs from VK*MYC mice had elevated levels of CD25. Tregs from MOPC-MM model upregulated the co-inhibitory receptors Tim-3, Lag-3, and TIGIT. **C** Double positive Tregs for ICOS/ICAM-1 and CD69/ICAM-1 increased in MOPC-MM. Double positive Tregs for Tim-3/Lag-3 and PD-1/Lag-3 emerged in the VK*MYC model. Median ± 95% CI of *n* = 5–13 mice, Mann–Whitney test *P ≤ 0.05, **P ≤ 0.01, ***P ≤ 0.001, and ****P ≤ 0.0001.

To investigate quantitative changes in Treg-number in the context of MM we used the previously described mouse model of MM-based on MOPC cells (MOPC-315.Luc-GFP.BMP3 in BALB/c mice) [19, 21]. Immunofluorescence staining of BM sections revealed that Tregs highly accumulated at sites of tumor growth in the BM of MM mice. Also, in areas with no visible tumor proliferation, the frequency of Tregs among CD4 T-cells and the density of Tregs increased compared with healthy mice (Fig. 1A and Supplementary Fig. 1A–C). To investigate local changes in the immune environment in myeloma patients, we analyzed BM aspirates of 55 myeloma patients, of which 30 were minimal residual disease (MRD) negative and 25 MRD positive at the time of analysis (patients’ characteristics in Supplementary Table 1). Treg frequencies in MRD positive patients exceeded those of MRD negative patients (Median: 12.8% vs. 9.2%, respectively, *P* = 0.074) (Fig. 1A). Moreover, the ratio of conventional T cells to Tregs was reduced in MRD positive compared to MRD negative patients (27.9 vs. 16.6, respectively, *P* = 0.061) (Supplementary Fig. 1D). We defined human Tregs by expression of FoxP3 and confirmed that 90% of these cells co-expressed CTLA-4 and the highest CD25 expression levels indicating a suppressive Treg population (Supplementary Fig. 1E–G). Based on these observations in myeloma patients and the MOPC mouse model we concluded that Tregs accumulate in direct vicinity within the MM-microenvironment and asked whether these interactions alter Treg activation determining their suppressive function.

### Tregs in MM are activated and display an effector phenotype

To estimate Treg activity in MM we analyzed the expression of Treg surface receptors at late disease stages in two immunocompetent MM models. Tregs from BM of MOPC-MM mice expressed higher activation marker levels of CD25, ICAM-1, CD69, and CD44 and coinhibitory receptors Lag-3, Tim-3, and TIGIT compared with healthy mice (Fig. 1B left), Also, in VK*MYC-mice (Vk12653 in C57BL/6) CD25 and CD69 expression increased, although less pronounced than in MOPC MM mice (right). Notably, co-upregulation of ICAM-1, ICOS, and CD69 in MOPC-MM and Lag-3, Tim-3, and PD-1 in VK*MYC MM indicated an even higher activation status (Fig. 1C). Except for CD25, MOPC-MM barely influenced the expression of activation markers on Tregs in the spleen, highlighting the role of the BM as the major tumor environment in this MM model (Supplementary Fig. 2 left). According to the higher tumor burden in the spleen in VK*MYC bearing mice, Tregs displayed here also a more activated phenotype (Supplementary Fig. 2 right). Notably, MM affected also conventional CD4^pos^ and CD4^neg^ T-cells changing their activation marker expression. As Tregs in both MM models were activated at sites of MM proliferation suggesting a supportive function in MM niche environments, we further elucidated the role of Tregs in functional in vivo experiments.

### Tregs impede an effective anti-MM response

**Fig. 2:**
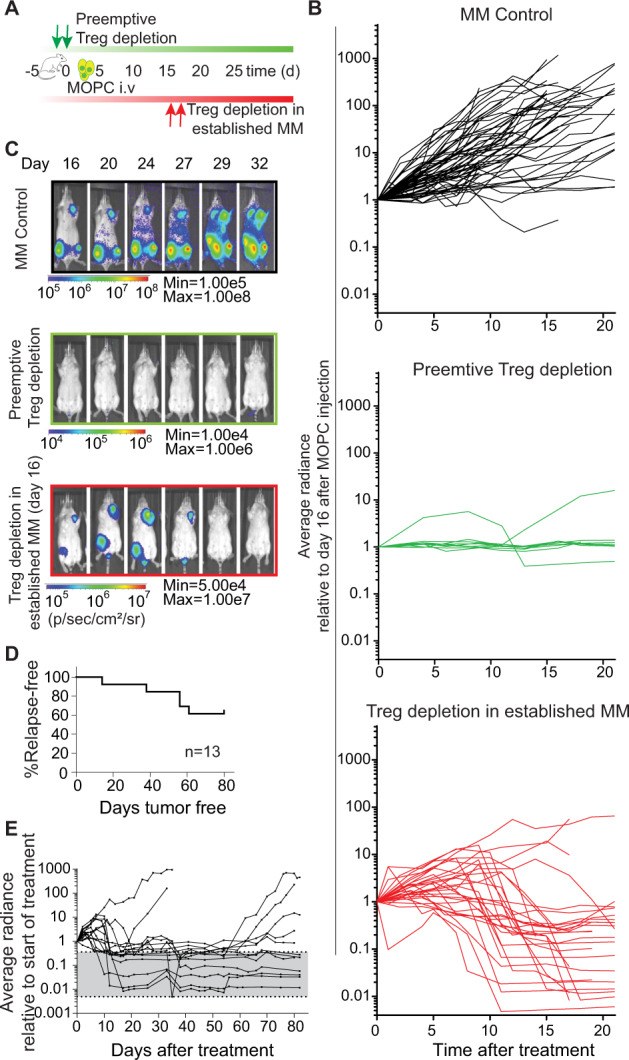
Tregs prevent an effective anti-tumor response. **A** Experimental setup: Tregs were depleted in DEREG mice by administration of diphtheria toxin (DTx) (20 ng/g body weight) on either day −1/0 prior to (green) or day 16/17 post (red) MOPC injection. **B**–**E** Disease progression was monitored with noninvasive BLI. **B** Average radiance normalized to individual signals at day 16 is shown. Each line represents tumor signal of an individual mouse. As control, both DEREG mice without DTx and WT mice plus DTx were used (black, *n* = 60). Preemptive depletion of Tregs prior to MOPC injection prevented engraftment of MM cells (green *n* = 10). Depletion of Tregs in mice with established MM resulted in an effective anti-tumor response with complete tumor remission (red, *n* = 29). **C** Tumor progression of one representative mouse per group **D, E** Long-term follow-up analysis after Treg depletion at day 16 in mice that were in complete remission. **D** Progression-free survival. **E** The BLI course of single mice is shown. The grey bar indicates the range of background signal of healthy mice normalized to the average signal on day 16. Mann–Whitney test: **P ≤ 0.01, ***P ≤ 0.001, and ****P ≤ 0.0001.

To specifically deplete Tregs in vivo we used C.DEREG mice [22]. Diphtheria toxin (DTx) selectively destroyed more than 70% of Tregs in BM and spleen three days after the first administration (Supplementary Fig. 3A). Notably, the Treg population recovered completely after 10 days in the BM and even faster in the spleen (Supplementary Fig. 3B). First, to investigate the role of Tregs in MM engraftment and dissemination, we depleted Tregs prior to MOPC injection (Fig. 2A). Tumor engraftment was completely inhibited in 9/10 of these mice (Fig. 2B, C). This emphasized the role of Tregs in engraftment and dissemination of MM to other BM loci.

**Fig. 3:**
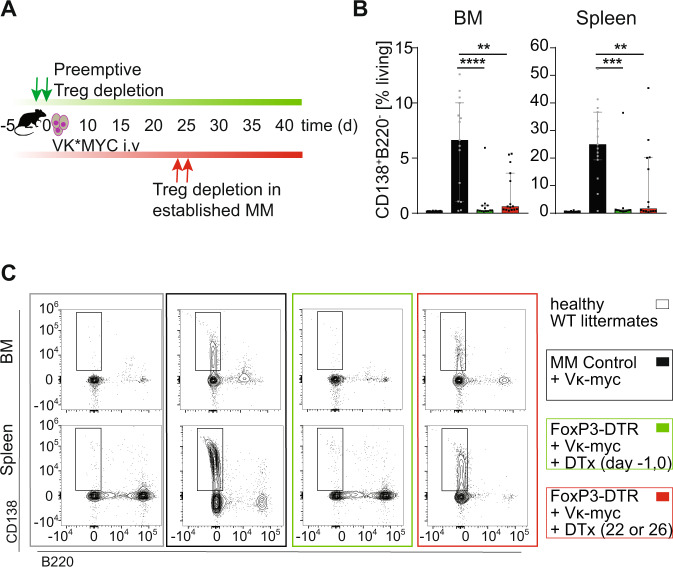
Tregs prevent an effective anti-tumor response in the VK*MYC-model. **A** Experimental set-up: VK*MYC cells were injected (i.v.) into B6a.FoxP3.Luci.DTR transgenic mice and Tregs were depleted by administration of diphtheria toxin (DTx) (20 ng/g body weight) on either day −1/0 prior to (green *n* = 13) or on two consecutive days between day 22 and 26 after VK*MYC injection (red *n* = 15). MM control mice were both WT littermates and B6a.FoxP3.Luci.DTR mice receiving either DTx or PBS (black *n* = 15). Tumor burden was determined at the end of experiment in BM and spleen with flow cytometry. **B** Quantification of CD138^+^B220^−^ cells within living mice. **C** Representative plots are shown (Median ± 95% CI of *n* = 10–15 mice). Mann–Whitney test: *P ≤ 0.05, **P ≤ 0.01, ***P ≤ 0.001.

**Fig. 4:**
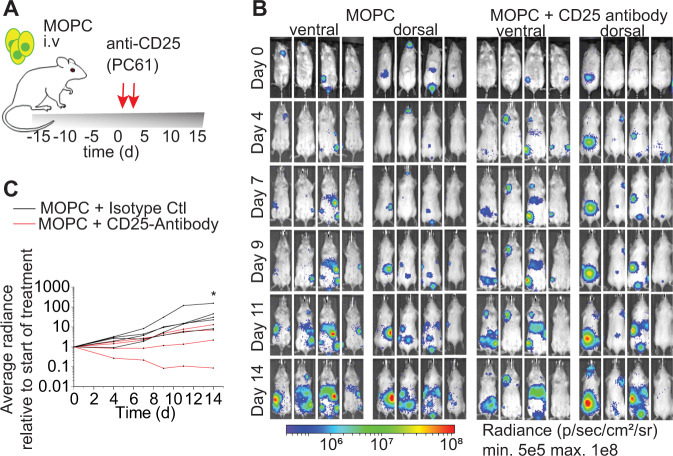
Anti-CD25-mediated Treg depletion mitigates tumor growth. **A** Mice with engrafted MM were treated twice with anti-CD25-antibody (100 µg/mouse/injection i.p.). **B** Tumor progression was monitored with non-invasive BLI. **C** Tumor growth relative to the signal at the start of the therapy. Two-way ANOVA with Sidak’s multiple comparisons test: day14 ^∗^*P* ≤ 0.05 (*n* = 4–5).

Second, to determine whether Tregs can serve as suitable therapeutic targets in established MM disease, we transiently depleted Tregs in mice with established MOPC-MM disease, which we had confirmed with non-invasive BLI before Treg depletion. Strikingly, almost all mice (27/29) lost tumor signal below the limit of detection within 16 days after short-term Treg depletion (Fig. 2B, C). Moreover, 9/14 mice remained tumor-free for an observation period of 80 days after Treg depletion, indicating continuous immune protection against MM. Five mice relapsed after 14 to 61 days with a median time of 56 days, all with recurrence of the BLI signal at locations distinct from primary tumor sites (Fig. 2D, E). To confirm this role of Tregs in an independent MM model we selectively depleted Tregs with DTx employing B6a.FoxP3.Luci.DTR mice [26] bearing VK*MYC MM (Fig. 3A). Control mice (WT littermates treated with DTx or B6a.FoxP3.Luci.DTR mice treated with PBS) developed a detectable and strong M spike after VK*MYC MM cell injection (in 14/15 mice) as measured with serum protein electrophoresis (Supplementary Fig. 4A). In all these controls tumor cells (defined as CD138^+^ B220^-^ cells with flow cytometry) appeared prominently in BM and spleen. Notably, VK*MYC MM cell numbers in the spleen exceeded tumor cell numbers in the BM by far (Fig. 3B, C). Depletion of Tregs prior to tumor cell injection prevented MM engraftment and we could detect M spikes only in 4/13 mice (Supplementary Fig. 4). MM infiltration of the BM in Treg-depleted mice was more than 7-fold (pre-emptive depleted) and 3.5-fold (depletion in established MM–day 22 or day 26 after VK*MYC injection) lower than in MM control mice (Fig. 3B, C). Similarly, the tumor burden in Treg-depleted mice was markedly reduced in the spleen (Fig. 3B, C). In contrast, the weight of spleens was increased in all MM control mice with macroscopically clearly visible metastasis, whereas this was only observed in single mice when we depleted Tregs pre-emptively or in established MM (Supplementary Fig. 4B). Taken together, also in the VK*MYC model, Tregs provided a supportive niche for MM engraftment and progression. These results support that Tregs can indeed serve as attractive therapeutic targets also in the treatment of MM. Consequently, we treated mice with established MOPC-MM with CD25-depleting antibodies. Indeed, depletion of CD25^+^ cells mitigated MM progression (Fig. 4). Yet, this strategy of CD25 targeting did not achieve a similar anti-MM effect as genetic Treg depletion, questioning whether this target would also impair anti-MM immune effector mechanisms. Besides, its constitutive expression on Tregs, CD25 can be found on different immune cells as diverse as activated effector T cells, memory T cells, NK cells, innate lymphoid cells, pre-B cells, dendritic cells, and oligodendrocytes. Therefore, it became clear that we needed to elucidate the immune effector mechanisms triggered by Treg depletion, which resulted in effective MM regression.

### Tregs inhibit effector cell functions in mice with MOPC-MM

**Fig. 5:**
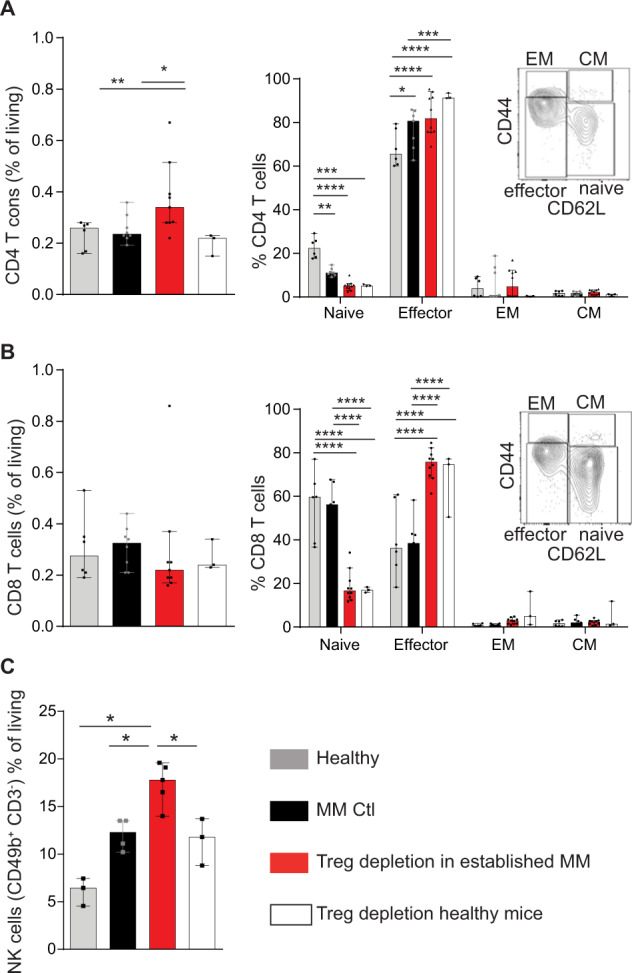
Effector T cells and NK cell numbers increase after Treg depletion in MM. T cells and NK cells were analyzed with flow cytometry in healthy mice, in MM bearing mice, and one week after Treg depletion in MM bearing and healthy controls. **A** CD4 Tcons increased in MM after Treg depletion. Within CD4 Tcons naive cells decreased and effector cells increased after Treg depletion in healthy and MM mice. **B** CD8 T cell frequency was not affected by Treg depletion, however, naive cells decreased and effector cells increased after Treg depletion in healthy and MM mice. **C** NK cells increased in MM, but further increased after Treg depletion. (Median ± 95% CI of *n* = 3–9 mice) Mann–Whitney test and two-way ANOVA with Tukey test for T cell subpopulations: *P ≤ 0.05, ** *P* ≤ 0.01, ***P ≤ 0.001, and ****P ≤ 0.0001.

**Fig. 6:**
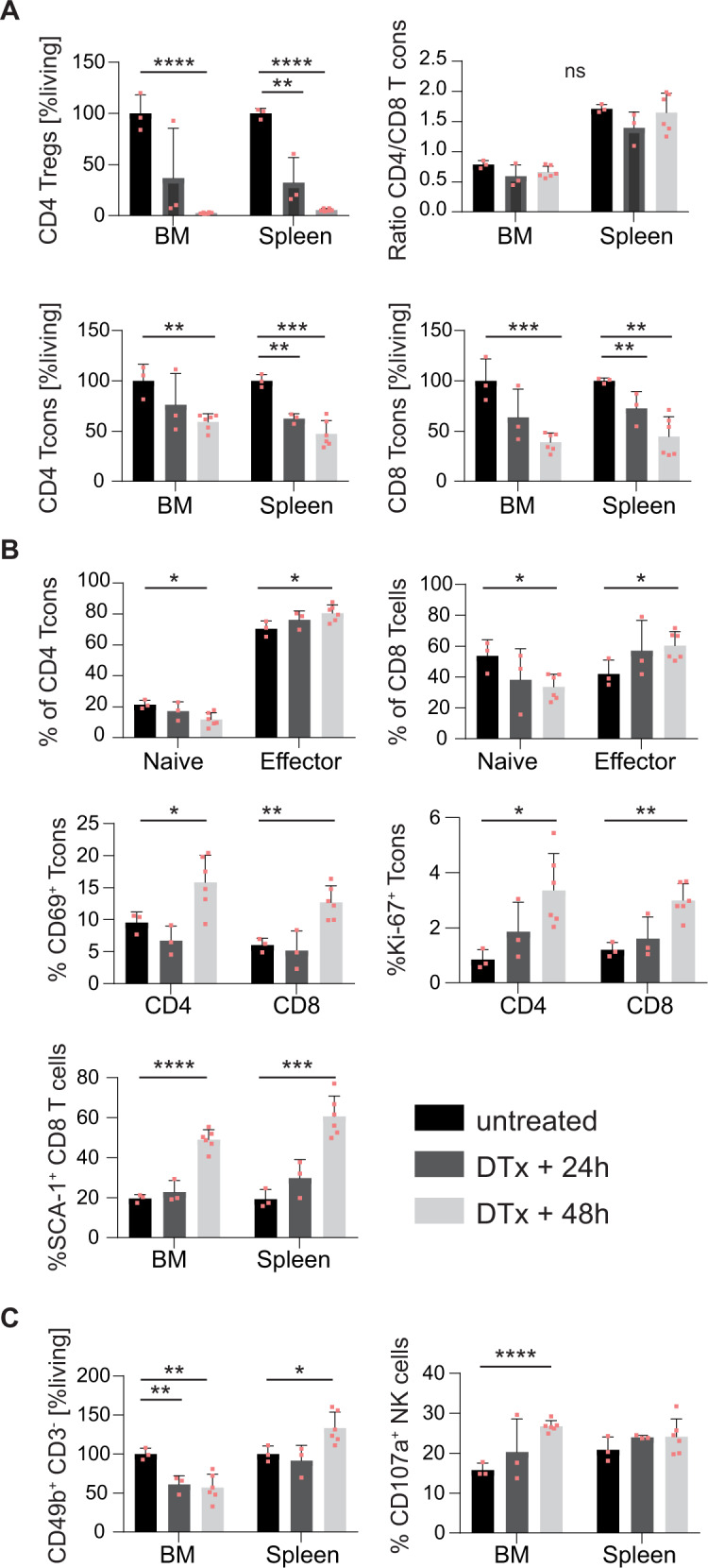
Rapid T cell activation after Treg depletion. T cells and NK cells were analyzed with flow cytometry in healthy, untreated DEREG mice (*n* = 3), 24 h (*n* = 3), and 48 h (*n* = 6) after DTx administration. **A** Tregs were efficiently depleted 48 h after DTx administration in the BM and spleen. DTx also reduces CD4 and CD8 T cons, but the ratio CD4/CD8 remains unchanged. Relative frequency to untreated. **B** CD4 and CD8 Tcons are already activated 48 h after DTx, shown by a shift from naïve (CD62L^+^CD44^low^) to effector phenotype (CD62L^−^CD44^pos^), increased CD69 expression, and a higher frequency of Ki-67 positive cells. SCA-1 was increased in CD8 T cells in BM and spleen. **C** The frequency of NK cells decreased but more NK cells expressed CD107a, indicating a higher rate of degranulation. Mean ± SD of *n* = 3–6, unpaired *t-*test: *P ≤ 0.05, **P ≤ 0.01, ***P ≤ 0.001, and ****P ≤ 0.0001.

Notably, in untreated MM mice, conventional T cells co-upregulated Tim-3 and Lag-3 suggesting T-cell exhaustion [29, 30] (Supplementary Fig. 5A). This tumor-induced dysfunction of conventional T cells was reversed in mice after Treg depletion and respective tumor regression, suggesting an invigoration of T-cell immunity. To investigate if conventional T cells serve as antitumoral effector cells after Treg depletion, we assessed changes in T-cell activation seven days after DTx-mediated Treg depletion in established MOPC-MM with flow cytometry. Conventional CD4 T cells increased over 1.5-fold in the BM after Treg depletion only in MM mice (Fig. 5A). In addition, CD4, and CD8 T cells shifted from a naive (CD62L^hi^ CD44^low^) to an effector (CD62L^low^ CD44^int^) phenotype. The overall CD8 T cell frequency in the BM did not change after Treg depletion (Fig. 5A, B). In MM, NK cells frequency increased in the BM and further increased after the depletion of Tregs, which also caused an increase in NK cells in healthy mice (Fig. 5C). Furthermore, more CD8 and NK cells produced granzyme B after the depletion of Tregs (Supplementary Fig. 5B). These results show, that CD4 and CD8 conventional T cells but also NK cells are activated seven days after Treg depletion and are potential effector cells after Treg depletion in established MM. To estimate the function of conventional T cells in the prevention of MM engraftment, we analyzed the kinetics of Treg depletion mediated conventional T cell activation 24 h and 48 h after DTx administration in healthy DEREG mice. DTx reduced not only Tregs, but also conventional CD4 and CD8 T cells within 48 h retaining the ratio between the subtypes (Fig. 6A). Nevertheless, conventional T cells displayed 48 h after DTx already an activated phenotype, shown by a shift in naïve to effector subset and an increased frequency of CD69^+^ and Ki-67^+^ cells (Fig. 6B). Furthermore, 48 h after DTx administration more CD8 T cells expressed SCA-1 in BM and spleen, a marker associated with T-cell activation and antigen-experienced memory T cells [31, 32]. Treg depletion reduced NK cell frequencies in the BM and increased in the spleen, but more NK cells expressed CD107a, indicating a higher degranulation rate (Fig. 6C). These data show that Treg depletion rapidly resulted in activation of conventional T cells and NK cells that rendered the BM environment hostile for MM engraftment and dissemination. Taken together, these results pinpointed CD4, CD8, and NK cells as potential effector cells against MM after Treg depletion. Therefore, we used cell-type-specific depleting antibodies to analyze these potential effector cells in vivo.

### CD8 and NK cells are main effector cells after Treg depletion

**Fig. 7:**
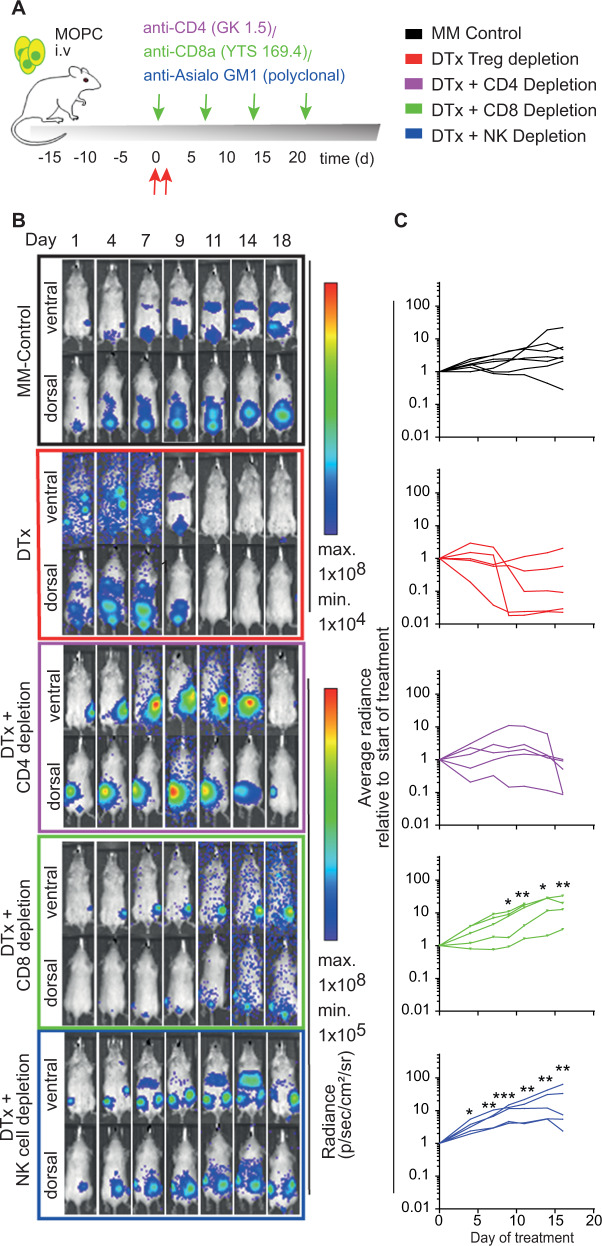
Depletion of Tregs unleashes CD8 and NK cell responses resulting in MM remission. **A** Experimental setup; Tregs were depleted in DEREG mice by administration of diphtheria toxin (DTx) at day 16/17 post-MOPC injection. In addition, starting with the Treg depletion, antibodies against CD4 (GK1.5 200 µg/week), CD8 (YTS169.4 initial injection: 400 µg, then 200 µg/week) or NK cells (anti-asialo-GM polyclonal, 35 µg/week) were given once weekly. **B**, **C** Disease progression was monitored with noninvasive BLI. **B** Representative BLI progression of one mouse/group. **C** Average radiance of *n* = 5–7 mice per group normalized to individual signals at day 16 is shown. Each line represents tumor signal of an individual mouse. Differences compared to DTx Treg depletion group: Kruskal–Wallis test with Dunn’s multiple comparisons and adjusted *p* values: *p ≤ 0.05, ** *p* ≤ 0.01, and ***p ≤ 0.001.

To selectively target potential immune effector cell populations, we first confirmed depletion efficacy with antibodies in the BM for CD4 (99.3%) and CD8 (99.3%). Anti-asialo-GM depleted 73–85% of NK cells (Supplementary Fig. 6). Notably, asialo-GM1 antibody did also reduce CD8 T cells by 60%. Subsequently, in mice with established MOPC-MM we depleted simultaneously Treg with DTx and CD4 or CD8 or NK cells in vivo (Fig. 7A). Mice depleted of CD4 T cells in addition to Tregs showed a delayed tumor regression compared with Treg depletion-control (Fig. 7B, C violet). However, 18 days after the administration of DTx and CD4-depleting-antibody, MM was in complete remission compared with mice only depleted of Tregs (Fig. 7B, C red). This emphasizes the role of CD4 T cells in the initiation phase of the anti-tumor response that is delayed but fully functional after CD4 T-cell depletion. In stark contrast, when we depleted either CD8 T cells or NK cells in Treg depleted mice, MM did not regress at all. Strikingly, in these mice, MM grew unhindered, similar, or even faster than in untreated mice. Therefore, both CD8 and NK cells are required for an effective tumor regression indicating a synergistic action of CD8 T cells and NK cells (Fig. 7B, C green/blue) as major effector cells against MM.

## Discussion

We show a fundamental role of Tregs in the engraftment and progression of MM in two independent immunocompetent mouse models. Strikingly, already a short-term reduction of Tregs sufficed for a long and stable remission of MM. Both, NK and CD8 cells were required for the effective anti-tumor response.

In recent years, the role of Tregs in MM has been discussed controversially in the literature [11–15, 18]. Here we show that Tregs accumulate in the region surrounding MM in the BM and display a highly active immune-suppressive phenotype. Moreover, we found that Tregs are enriched in myeloma patients after treatment when MRD positive, compared to a reduced frequency in MRD negative patients. This further highlights the role of Tregs in the progression of MM and is in alignment with recent findings [33]. The frequency and activation of Tregs in MM have been investigated in several studies with different outcomes. Kawano et al. found an increase in Tregs in the BM of VK*MYC mice and in BM aspirates of MM patients [18], whereas Foglietta et al. did not find any differences in Treg frequency in the BM of MM patients [11]. In the 5T2MM mouse model, an increase of Tregs was only seen at a late stage of disease [34]. Based on flow cytometry we also did not observe an altered overall abundancy of Tregs in BM of MOPC MM mice. In contrast, analyzing single bones separately with immune fluorescence microscopy revealed an accumulation of Tregs especially in regions of MM growth. Increased demand of space of progressing MM might displace Tregs to adjacent areas resulting there in a local enrichment of Tregs. These local differences in Treg accumulation may at least partly explain apparent contradictions in different studies because based on our observation, local tumor burden and distribution define whether changes in Treg numbers can be found.

Kawano *et al*. showed in the VK*MYC mouse model, that mice constantly depleted of Tregs for weeks after tumor injection are protected from MM engraftment and survive longer [18]. However, continuous depletion of Tregs results ultimately in severe autoimmunity [35]. To assess a potential therapeutic effect, we short-term depleted Tregs in mice with already established MM disease. We demonstrated that the progression of MM depends on the presence of Tregs. In the MOPC model, mice were completely protected from MM growth already after a single depletion of Tregs before tumor cell injection without continuous depletion. Moreover, in mice with established MOPC-MM a single depletion of Tregs resulted in an effective and persistent immune response against MM. This effect is especially striking, as Treg numbers had already recovered within ten days after depletion. Therefore, it is even more remarkable, that almost all mice stayed tumor-free for the entire observation period of 80 days. Zaretsky et al. showed that Tregs and plasma cells colocalize in the BM and that depletion of Tregs reduces the amount of non-malignant plasma cells in the BM of healthy mice [36]. This protective niche might be abused by malignant plasma cells and the disruption of this niche might contribute to the tumor regression. Even though we used adoptive tumor cells transfer models in which the immune system does not co-evolve with the tumor progression and induced immune tolerance towards the tumor might therefore be reduced, both models engraft in fully immunocompetent mice reflecting a highly aggressive and progressed (in the case of VK*MYC also Bortezomib resistant) MM disease. The long survival of mice excludes other conceivable reasons for the absent BLI signal, like clonal loss of luciferase expression. Strikingly, when we rechallenged two mice 100 days after initial MM injection, they were protected from MOPC-MM engraftment. This indicates persistent immune protection and anti-tumor immune memory formation, which occurred only when Tregs had been depleted at day 16. In contrast, 3/4 mice that were pre-emptively depleted of Tregs and rechallenged after 100 days showed MOPC engraftment and progression comparable to non-Treg-depleted mice. Therefore, we conclude that while Tregs essentially contribute to microenvironments permissive for MM engraftment, direct MM clearance before tumor cells could engraft prevented protective memory formation. Vice versa, short-term Treg depletion in mice with established MM allowed for protective anti-tumor immune memory formation.

CD8 T cells and NK cells are potent killers of tumor cells [37, 38]. Importantly, independent of the disease stage, MM cells remain highly susceptible to cytotoxic killing by T and NK-T cells [39, 40]. Considering the enormous potential of engaging cytotoxic immune effector mechanisms against MM, a rich pipeline of CAR-T cells and T cell engaging antibodies against diverse MM targets as well as NK cell therapies are currently explored [41–46]. Nevertheless, conventional T cells reportedly display an exhausted and senescent phenotype in myeloma, executing only reduced effector functions [47–49]. In line with these findings, we also observed an exhausted T cell phenotype with a high co-expression of Tim-3 and Lag-3 on conventional T cells in our murine models. Strikingly, this can be completely reversed by short-term and transient Treg depletion. Treg depletion resulted in the strong activation of conventional T cells and NK cells. Interestingly, T cells and NK cell activation occurred within 48 h after Treg depletion highlighting the vast immunosuppressive capacity of Tregs. In line with this, we show that depletion of Tregs prior to tumor cell injection completely prevents tumor engraftment, presumably because of elevated immune surveillance of activated immune effector cells. Even though there are other immune-suppressive cells like myeloid-derived suppressor cells, the Treg mediated suppression appeared sufficient to restrain an otherwise very effective CD8 and NK cell response. Notably, the sole depletion of NK cells or CD8 T cells resulted in a CD226 dependent faster tumor progression in the VK*MYC MM model showing an ongoing attempt of immune control even under tumor progression [50]. We also observed an increased tumor progression when we depleted CD8 T cells or NK cells in addition to Tregs. Together with the CD8 T cell activation and the enhanced granzyme b secretion of CD8 T cells and NK cells after sole Treg depletion, this data argues for suppression of these effector cells by Tregs. However, the tumor progression after a loss of NK and CD8 T cell control might also disguise other possible anti-tumor effector cells suppressed by Tregs. Noteworthy, depletion of NK cells with anti-asialo-GM1 also reduced the number of CD8 T cells by 65%. Therefore, the lost anti-tumor effect after asialo-GM treatment shown here in this study can be caused by the additive effect of the depletion of NK cells and NK-T cells and the loss of CD8^asialoGM+^ T cell subset, which reportedly can induce allograft rejection [51]. As in MM CD8 T cells and NK cells are involved in the control of the disease [39, 40], and the dysfunction of these effector cells correlates with MM disease progression [50, 52, 53].

MM hampers the equilibrium of immunosuppression and activation provided by Tregs towards the immunosuppression. Strategies to target Tregs to re-balance the equilibrium appear attractive and promising for tumor therapy; however, they also bear the risk of autoimmunity. Notably, we did not observe any signs of autoimmunity besides MM eradication after transient Treg depletion. Thus, the profound effect of a short-term depletion of Tregs gives an advantage for therapeutic interference because it avoids autoimmunity caused by a continuous reduction of Tregs. CD25, the high-affinity receptor for IL-2 is constitutively expressed on Tregs and only transiently upon activation on conventional T cells. Treatment with CD25-depleting antibodies depletes Tregs in mice [54]. Interestingly, CD25 is also expressed on leukemia cells in some patients [55], and depleting CD25 antibodies are already approved for clinical use and, therefore, appear as a promising therapeutic target [56, 57]. However, in MOPC-MM mice we showed only a partial beneficial effect. Especially the low depletion efficacy in the BM and the unintentional depletion of freshly activated effector cells likely diminishes the benefit. Accordingly, targeting Tregs via CD25-antibody (Daclizumab) or by a DTx-IL2-fusion protein (ONTAK) did not benefit melanoma patients due to effector cell elimination [58, 59]. Non-IL-2 blocking anti-CD25 depleting antibodies (NIB) might be a promising option, as they combine similar Treg depletion efficacy with an increased effector response [60]. Considering the powerful effect to complete remissions by activating an endogenous CD8 T cell and NK cell-mediated immune response upon transient Treg depletion in our independent genetic models in MM bearing mice, emphasizes that strategies to target Tregs more selectively are desperately needed, and existing strategies must be reevaluated regarding the local bioavailability and depletion efficiency in the BM.

## Supplementary information

Supplemental information
