# Supplementary material for: Transient regulatory T-cell targeting triggers immune control of multiple myeloma and prevents disease progression
Source: Leukemia. 2021 Sep 28;36(3):790–800. doi: 10.1038/s41375-021-01422-y (PMC8885410; doi:10.1038/s41375-021-01422-y)
Supplement: Supplementary file 1 — Supplemental information [file 41375_2021_1422_MOESM1_ESM.pdf]

Supplemental information

Online supplemental material:

Fig. S1 shows that Tregs are highly abundant in areas of myeloma tumor growth. Fig. S2 shows the expression of activation markers on Tregs and conventional T cells in BM and spleen of MOPC and VK*MYC MM mice. Fig. S3 shows Treg depletion efficacy after DTx and CD25 antibody administration. Fig. S4 shows results from serum protein electrophoresis from all VK*MYC injected mice 35 days after tumor injection. Fig. S5 shows reduction of exhaustion marker on Tcons after Treg depletion in MM. Fig. S6 shows the depletion efficacy after CD4/CD8 and asialo-GM1 antibody-mediated depletion in healthy mice. Supplemental methods describe applied statistics and general methods. Table S1 shows MRD patients' characteristics and Table S2 lists the antibodies used.

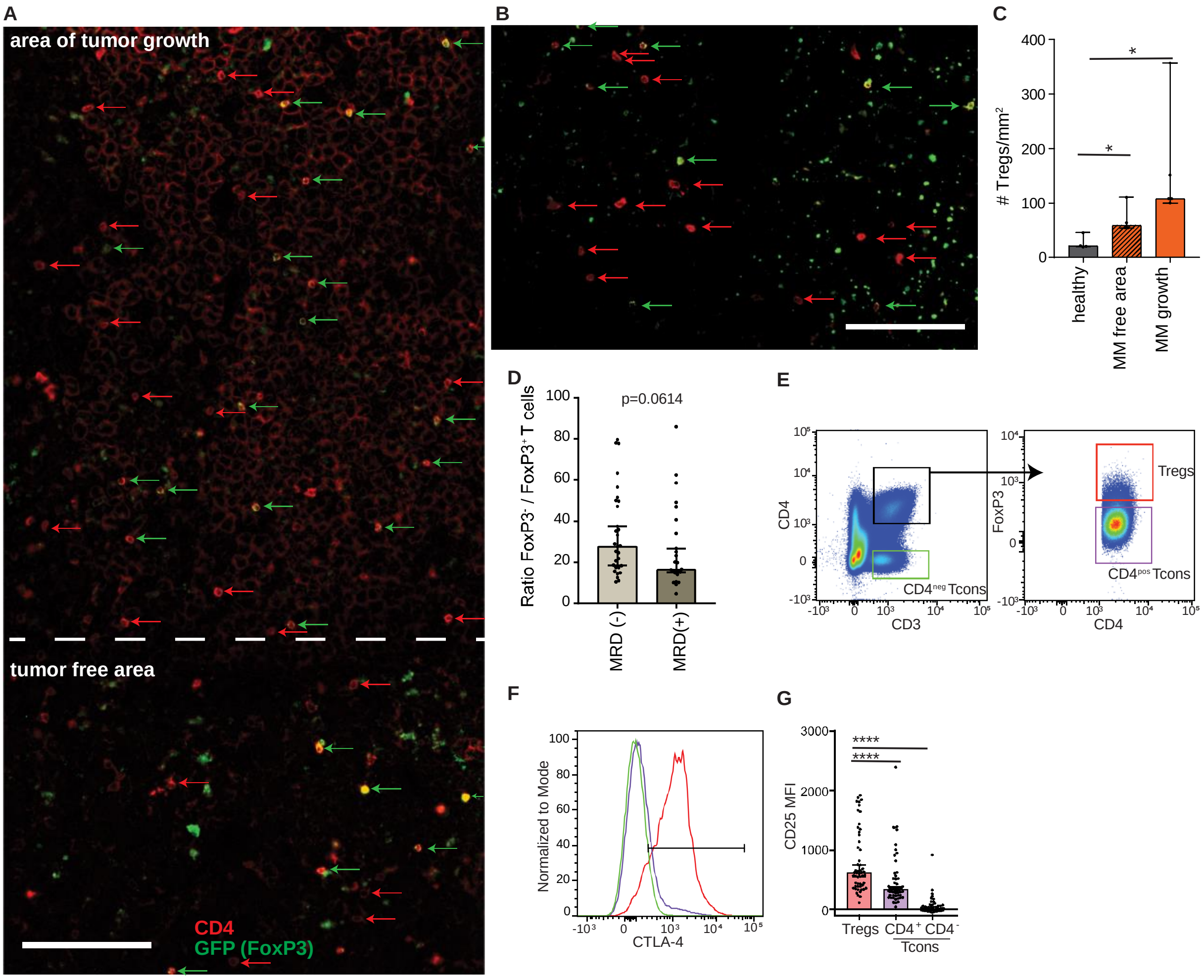

Supplemental Figure 1: Tregs are highly abundant in areas of myeloma tumor growth

(A) Longitudinal section of a femur from a MM bearing and **(B)** healthy C.DEREG mouse. Red: anti-CD4, green: anti-GFP (Tregs), red arrows indicate GFP^{neg} CD4 T cells and green arrows indicate GFP^{pos} CD4 Tregs. Larger CD4^{dim} cells in upper part are MOPC cells. Scale bar: 100 µm. **(C)** Density of Tregs was highest in mice with MM in areas of tumor growth**.** Areas were calculated using ImageJ and cells were counted manually. Median ± 95% CI of n=3-4**,** Mann-Whitney test **P≤0.05.* **(D)** Ratio of FoxP3^{neg} conv. T cells (CD3^{pos}CD4^{pos} + CD3^{pos}CD4^{neg}) to FoxP3^{pos} Tregs was reduced in MRD positive compared to MRD negative myeloma patients. Median ± 95% CI of n=25 MRD positive, n=30 MRD negative, n=2 healthy controls, Mann-Whitney test *p=0.0614*. **(E)** Gating strategy of Tregs from BM aspirates of myeloma patients (pre-gated on singlets and living cells). **(F)** 90% of FoxP3^{pos} Tregs express CTLA-4 (exemplary histogram; red: Tregs, violet: CD4^{+}Tcons, green CD4^{-}Tcons, n=2) **(G)** Highest expression of CD25 (MFI) on FoxP3^{pos} Tregs compared to CD4^{+}Tcons and CD4^{-}Tcons of myeloma patients, n=54 BM aspirates

of MRD positive and negative myeloma patients. Median ± 95% CI, Mann-Whitney test **** *p<0.0001.*

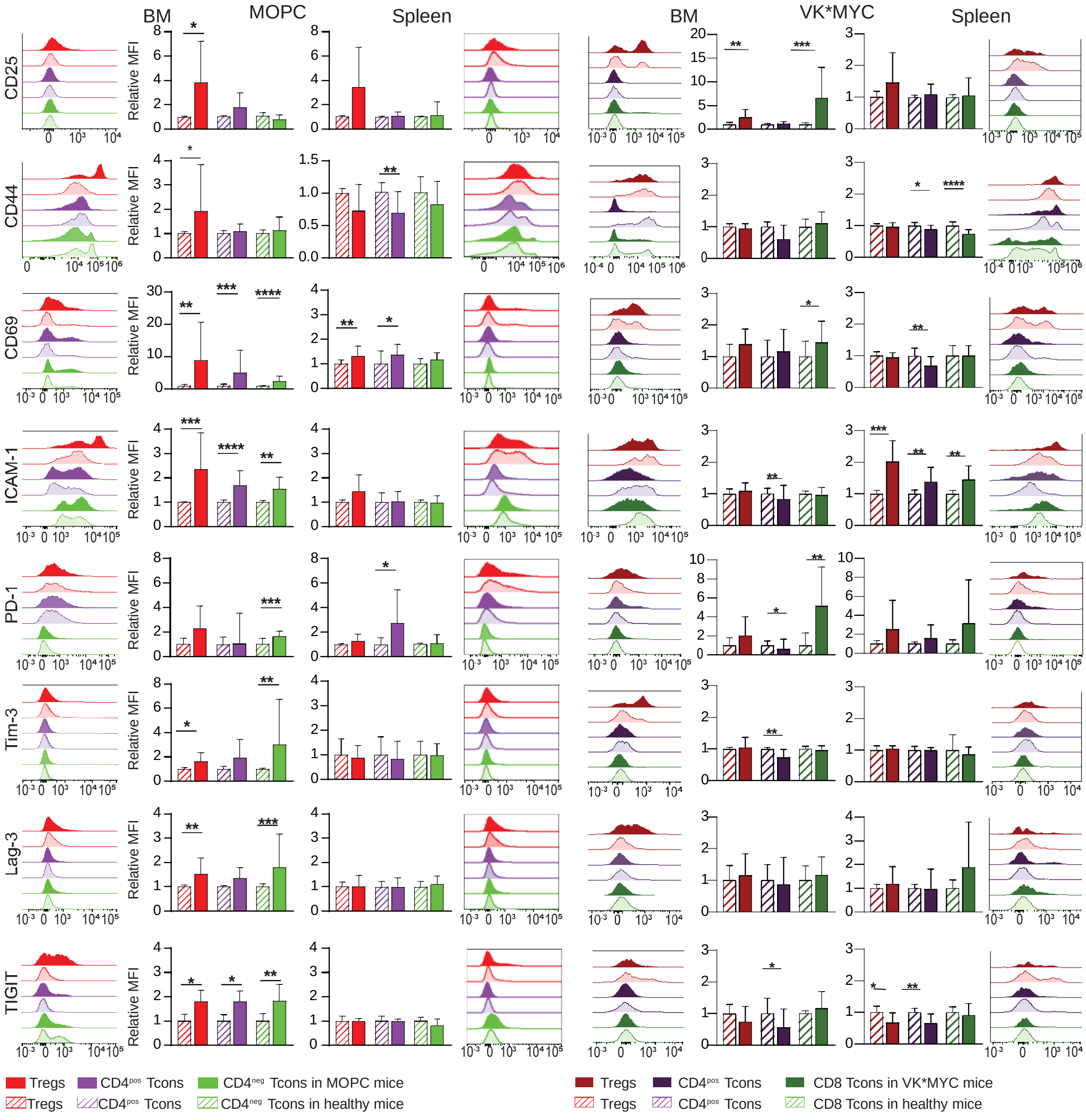

Supplemental Figure 2 T cell activation restricted to the BM niche in MOPC-MM mice, and both Tregs and conventional T cells upregulate activation markers

Expression of activation marker and co-inhibitory receptors on Tregs, CD4^{pos} and CD4^{neg} T cons. Expression on different T cell subsets is shown with one representative flow cytometry histogram for BM and spleen. Mean fluorescence intensity of receptors on Tregs (red), CD4^{pos} Tcons (purple) and CD4^{neg} /CD8 T cons (green) in MOPC-MM mice (uni-color - columns, dark tinted - histograms)

relative to the respective population in healthy littermates (striped pattern - columns, bright tinted - histograms). N=5-12 mice were analyzed.

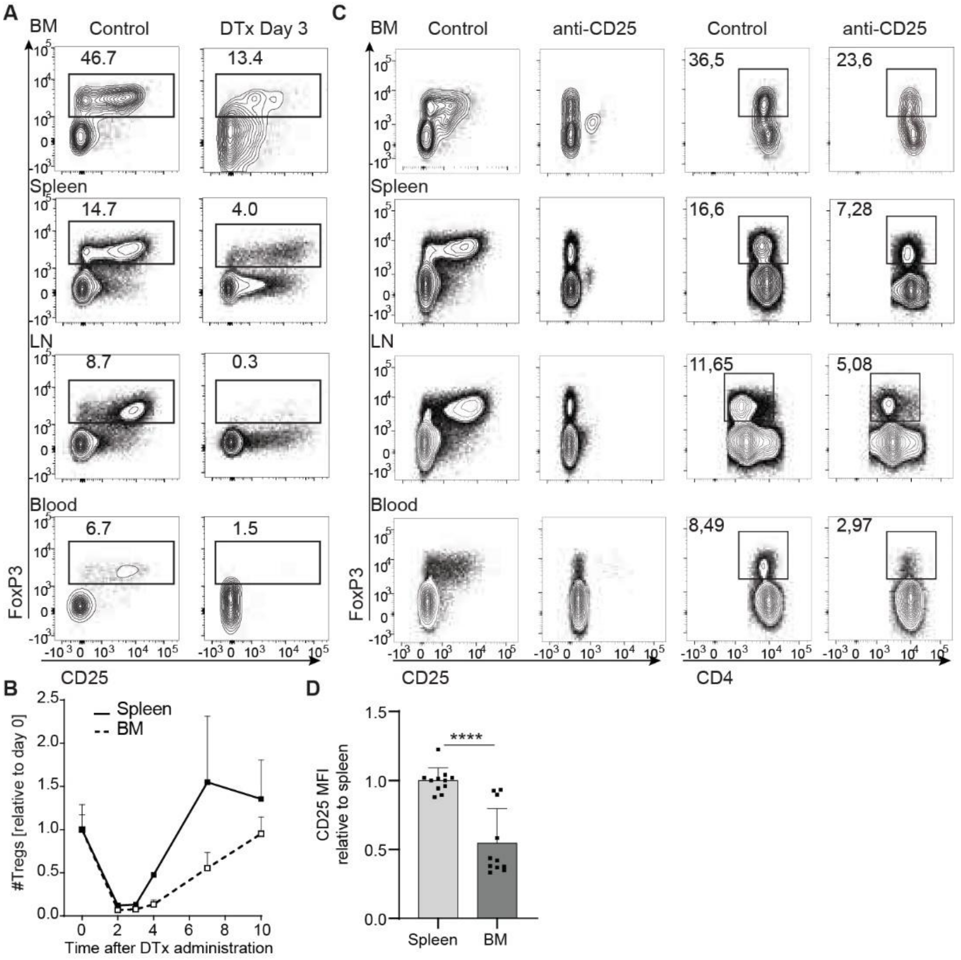

Supplemental Figure 3 Efficient and transient depletion of Tregs in DEREG mice

(A) Tregs were depleted with DTx (20 ng/g bodyweight) in DEREG mice on two consecutive days. One representative plot at day 3 after DTx is shown. Tregs are defined by CD4 and FoxP3 expression **(B)** Recovery of Treg number after DTx administration on day 2,3,4, 7 and 10 (N=2-3 per timepoint) **(C)** BALB/c Mice received 6×100 µg CD25-antibody (PC61) i.p. every other day and organs were analyzed 7 days after last administration. Depletion efficacy is indicated with one representative flow cytometry plot per organ (N=2). **(D)** Expression of CD25 is reduced on Tregs of the BM compared to spleen. Mann Whitney test ****P<0.0001.

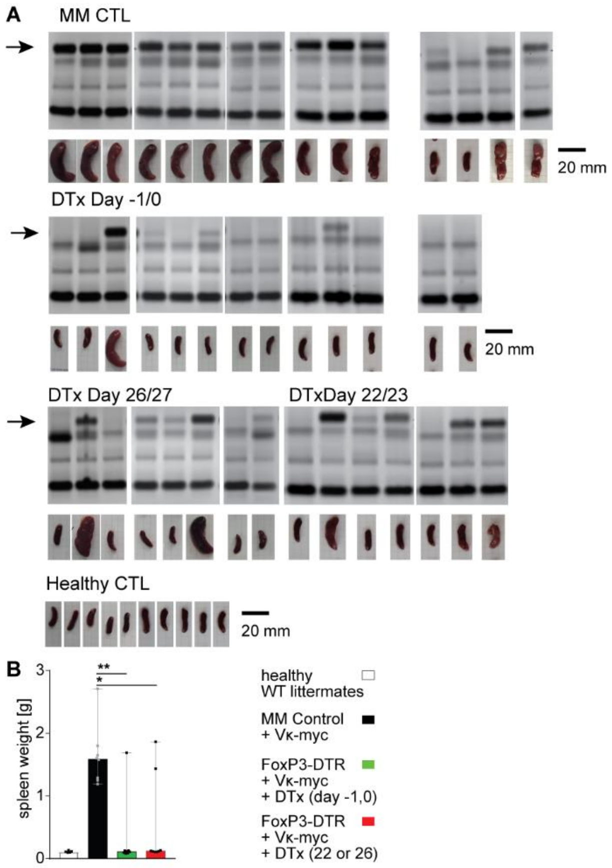

Supplemental Figure 4 Reduced tumor burden after Treg depletion in VK*MYC mice

(A) Serum protein electrophoresis at day 35 after VK*MYC injection in control mice (n=15), compared to mice receiving DTx before tumor cell injection (n=13) and mice receiving DTx in progressed myeloma (n=15). Arrow indicates size of M-protein. Strong M-spike correlates with enlarged spleens with visible tumor burden at experiment end. **(B)** Weight of spleens was increased in MM control mice compared to both Treg-depleted groups (n=8) and healthy mice (n=5). Median ± 95 % CI, Mann-Whitney test: * P≤0.05 ** P≤0.01.

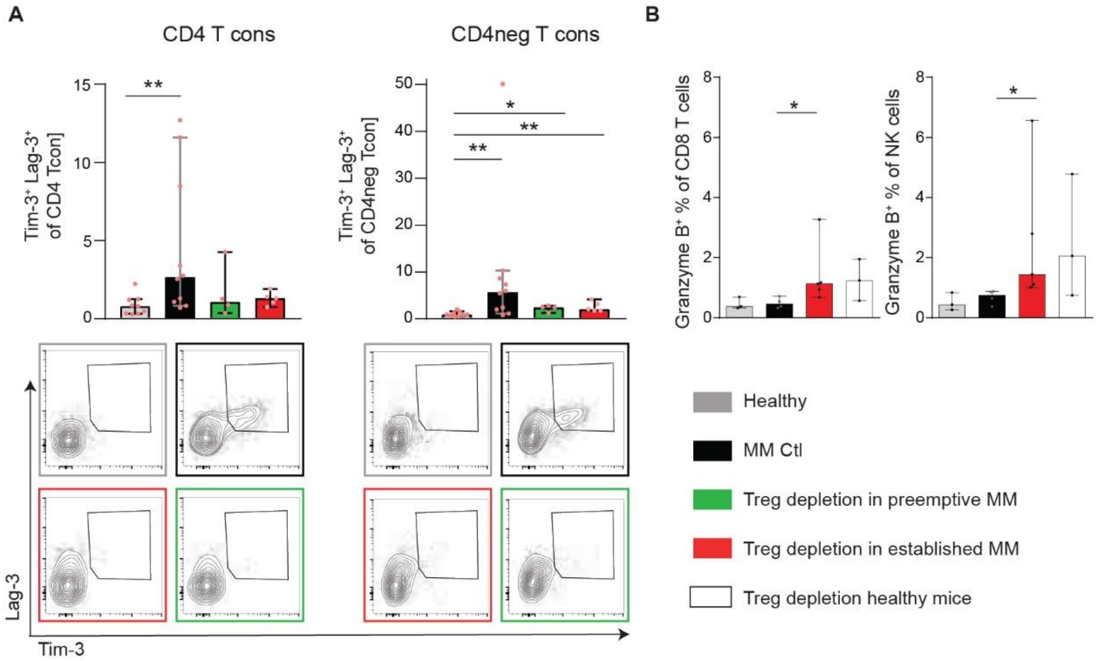

Supplemental Figure 5 Tcons reduce expression of exhaustion marker after Treg depletion in MM

(A) In MM (n=9) CD4 positive (left) and negative (right) Tcons co-upregulated the exhaustion markers Tim-3 and Lag-3. After Treg depletion (both, pre-emptive n=4 and in established MM n=5) Tim-3/Lag-3 expression approximated healthy controls (n=9). **(B)** More CD8 T cells and NK/NKT cells produce granzyme B after Treg depletion in healthy (n=3) and MM mice (n=5), compared to untreated healthy (n=3) and MM mice (n=4). Median ± 95 % CI, Mann-Whitney test **P≤0.05, ** P≤0.01*.

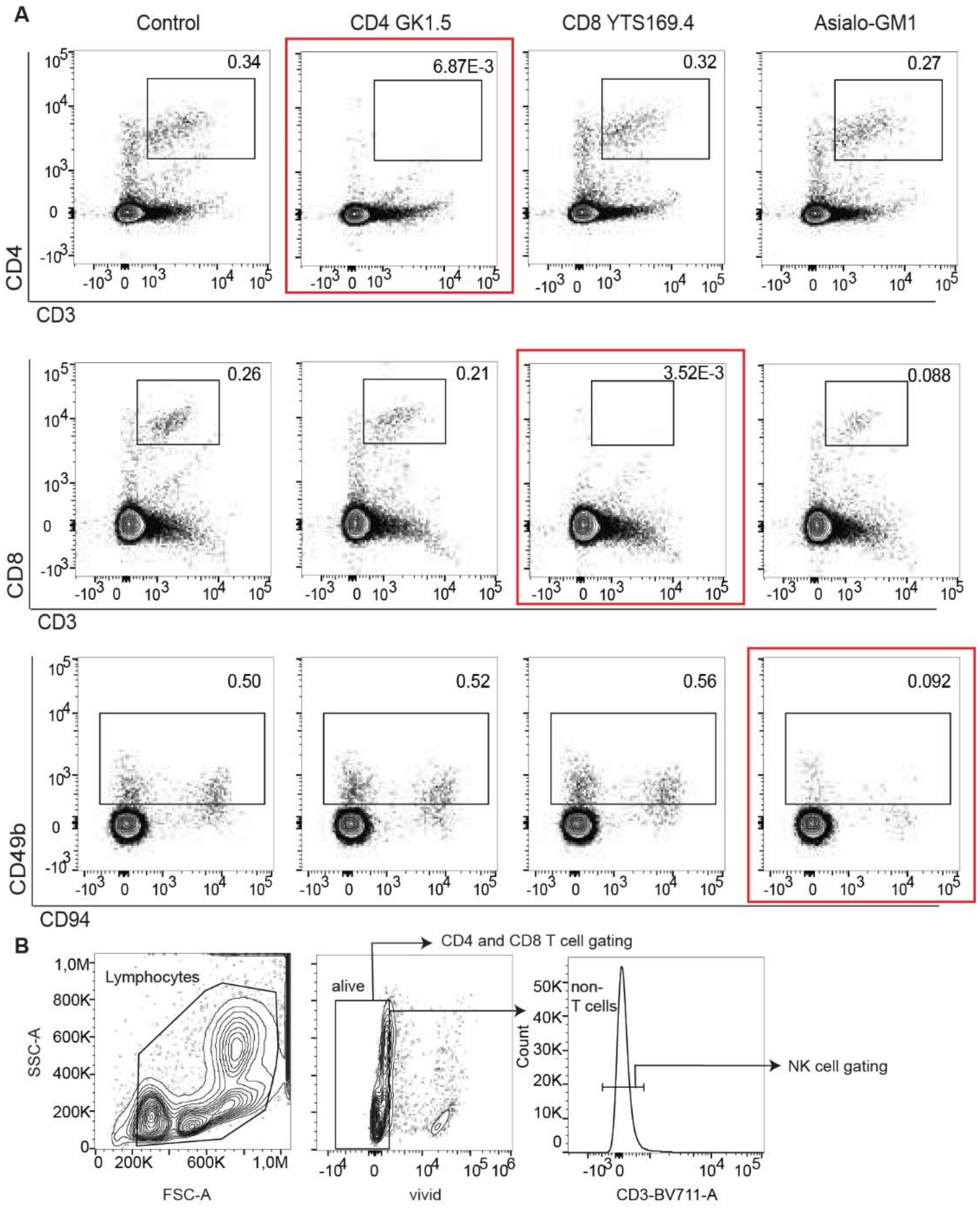

Supplemental Figure 6 Antibody-mediated depletion of target cells is efficient in the BM

BM was analyzed with flow cytometry five days after a single i.p. injection of anti-CD4 (GK1.5 - 200 µg/mouse), anti-CD8 (YTS 169.4 - 400 µg/mouse) or anti-Asialo-GM1 (35 µg/mouse) antibodies. (A) FACS blots of CD4 and CD8 T cells and NK cells with respective frequency are shown. (B) CD4 and CD8 T cells are gated on living lymphocytes and T cells are excluded from NK cells.

Supplemental methods:

Statistic and general methods:

Sample sizes of animal studies were approved by the Bavarian government. For the calculation of sample size and animal number the program GPower3.1.2 was used. Type 1 error was set to 5% and type 2 error to 20%. The expected effect size according to Cohen’s d was calculated using estimated differences in mean ±SD. To ensure an equal distribution of tumor burden and variation among groups, tumor mice were grouped according to the BLI signal (if applicable) before treatment. Mice without tumor burden were excluded at this time. Apart from different treatment (e.g., antibody or DTx administration) mice between groups were handled identical and analysis was constant between groups however the investigator was not blinded to the groups. For most data gaussian distribution or similar variance among groups was not given and non-parametric tests (e.g., Mann-Whitney or Kruskal-Wallis) were performed. P values are two-tailed and adjusted for multiple comparisons if applicable.

Table S1

Clinical characteristics of multiple myeloma patients

**General**		n	%
All patients		55	
Male		38	69.1
Female		17	30.9
Age at diagnosis, mean years (range)		60.9 (34-80)	
**International staging system (ISS) at diagnosis**			
I		21	38.2
II		13	23.6
III		8	14.5
ISS unknown	thereof Durie and Salmon staging	13	23.6
	II	1	
	III	4	
	staging unknown	7	12.7
**Myeloma Subtype**			
IgG		39	70.9
IgA		8	14.5
IgM		1	1.8
Light chain Bence-Jones		5	9.1
Non secretory or oligosecretory		2	3.6
kappa		20	36.4
kappa light		12	21.8
lambda		11	20.0
lambda light		8	14.5
Combination		3	5.5
Non secretory		1	1.8
Bone Lesions		13	23.6
Information not available		8	14.5
**Genetic Risk**			
High Risk		10	18.2
Standard Risk		45	81.8
**Disease Stage**			
PR		2	3.6
VGPR		16	29.1
CR		15	27.3
sCR		15	27.3
PD		6	10.9
SD		1	1.8
MRD negative		30	54.5
MRD positive		25	45.5
Alive Patients		55	100.0

Abbreviations: ISS = International Staging System, PR = partial response; VGPR = very good partial response; CR = complete remission; sCR = stringent complete remission; PD = progressive disease; SD = stable disease

Table S2

List of antibodies

target	clone	vendor	catalog #
anti-mouse:			
CD3	17A7	Biolegend	100241
CD4	GK1.5/RM4-5	Life Technologies	A15384
CD8	53-6.7	Life Technologies	56008182
FoxP3	FJK-16s	eBiosciences	53577382
PD-1	RMP1-30	Biolegend	109110
Lag-3	eBioC9B7W	eBiosciences	12223182
CD25	PC61.5	eBiosciences	45-025182
CD44	IM7	Biolegend	103012
CD69	H1.2F3	Life Technologies	250691182
ICAM-1	YN1/1.7.4	Biolegend	116120
Tim-3	8B.2C12	eBiosciences	17587182
TIGIT	GIGD7	eBiosciences	25950182
CD49b	Dx5	Life Technologies	56597180
Granzyme B	NGZB	eBiosciences	25889882
immunefluorescence microscopy			
CD4 (unlabeled, rat)	H129.19	BD Pharmingen	550278
GFP (unlabeled, rabbit)	polyclonal	Life Technologies	A11122
CD138 (APC, rat)	281-2	Biolegend	142506
anti-human			
CD3	OKT3	Biolegend	317314
CD4	OKT4	Biolegend	317428
CD4	S3.5	Life Technologies	MHCD0417
FoxP3	PCH101	eBiosciences	11477642
CTLA-4	eBio20A	eBiosciences	12152842
CD25	M-A251	Biolegend	356108
